# Psychometric Properties of Scale to Assess the Therapeutic Relationship—JapaneseVersion (STAR-J)

**DOI:** 10.3389/fpsyt.2019.00575

**Published:** 2019-09-18

**Authors:** Asami Matsunaga, Sosei Yamaguchi, Utako Sawada, Takuma Shiozawa, Chiyo Fujii

**Affiliations:** ^1^Department of Community Mental Health and Law, National Institute of Mental Health, National Center of Neurology and Psychiatry, Tokyo, Japan; ^2^Kitamura Institute of Mental Health Tokyo, Tokyo, Japan; ^3^Department of Psychiatric Nursing, Graduate School of Medicine, The University of Tokyo, Tokyo, Japan; ^4^Department of Nursing Sciences, Graduate School of Human Health Sciences, Tokyo Metropolitan University, Tokyo, Japan

**Keywords:** therapeutic relationship, scale, factor structure, reliability, validity, community mental health

## Abstract

**Background:** A good therapeutic relationship between patient and psychiatrist is vital for effective mental health care. However, no instruments to assess this relationship are available in Japan. This study aimed to develop a Japanese version of a Scale To Assess Therapeutic Relationship (STAR-J), which measures such relationships from the viewpoints of both the patient (STAR-J-P) and clinician (STAR-J-C). We examined the tool’s psychometric properties, including factor structure, internal consistency, convergent validity, and test-retest reliability among psychiatric outpatients and psychiatrists.

**Methods:** Study participants comprised 139 outpatients and 10 psychiatrists. Exploratory factor analysis was conducted to investigate factor structure; to confirm cross-validity, confirmatory factor analysis was conducted using a different sample constituting 195 participants in an assertive community treatment program and their 91 case managers. Cronbach’s alpha was used to assess internal consistency. For STAR-J-P only, the intra-class correlation coefficient (ICC) was computed for 17 patients to determine test-retest reliability. Spearman’s correlation coefficients were calculated to examine convergent validity with service satisfaction, empowerment, and medication adherence.

**Results:** We identified a two-factor structure for STAR-J-P and a one-factor structure for STAR-J-C. Cronbach’s alphas for the two STAR-J-P factors were 0.897 and 0.645, and that for the STAR-J-C factor was 0.949. The ICCs for STAR-J-P factors 1 and 2 were 0.765 and 0.630, respectively. STAR-J-P and STAR-J-C were not significantly correlated. STAR-J-P factors 1 and 2 showed significant correlations with service satisfaction (factor 1: ρ = 0.648, p < 0.001; factor 2: ρ = 0.238, p = 0.005) and medication adherence (factor 1: ρ = 0.508, p < 0.001; factor 2: ρ = 0.347, p < 0.001), but only factor 1 showed a significant relationship with empowerment (ρ = 0.283, p = 0.001). STAR-J-C was significantly correlated only with empowerment (ρ = 0.207, p = 0.017).

**Conclusions:** STAR-J appears to be a useful instrument for assessing therapeutic relationships in the Japanese psychiatric outpatient setting. Further studies should test its validity and applicability in different mental health service settings.

## Introduction

Over the past two decades, the subjective therapeutic relationship between psychiatric patients and clinicians has gradually become a meaningful outcome in mental health treatment (1), and the assessment of patient evaluations of the benefits of treatment has been important in mental health services (2, 3). The therapeutic relationship consists of the connections and interactions that occur between patients and clinicians during the delivery of mental health treatment (4). This relationship is assumed to be a core component of mental health care (5), particularly as increasing attention has been paid to recovery-oriented approaches in the field of mental health services (6), which require that clinicians build good relationships with their patients. Developing a good therapeutic relationship is not only the competency and skills that clinicians are expected to possess in order to improve quality of mental health services, it is also needed to develop better outcomes. For example, a good patient-clinician relationship has been found to be important not only for decision-making, but also for developing better patient-reported outcomes such as service satisfaction (7), empowerment (8), medication adherence (9, 10), and other clinical outcomes (11) in mental health service settings worldwide.

These findings indicate that evaluating the patient-clinician therapeutic relationship has become increasingly essential for assessing the quality and effects of mental health services. However, to the best of our knowledge, such instruments have not been available in mental health care settings in Japan. In the United Kingdom, the Scale To Assess Therapeutic Relationship (STAR) was developed in a community mental health service setting by McGuire-Snieckus et al. (12). This scale allows the patient and clinician to assess their subjective therapeutic relationship (STAR Patient version: STAR-P, and STAR Clinician version: STAR-C). In this study, we aimed to develop a Japanese version of STAR (STAR-J) and to examine its psychometric properties, including factor structure, internal consistency, test-retest reliability, and convergent validity among outpatients and psychiatrists in outpatient service settings.

## Materials and Methods

### Design and Settings

We conducted a cross-sectional questionnaire survey in an outpatient setting at one psychiatric hospital located in Tokyo and two psychiatric clinics located in Tokyo and Chiba, Japan, to assess the factor structure, convergent validity, and internal consistency of STAR-J-P and C, and the test-retest reliability of STAR-J-P. Ten psychiatrists were asked to participate in this study. Patient eligibility criteria were as follows: 1) receiving outpatient care from the psychiatrists who participated in this study, 2) aged 20 years or older, and 3) taking prescription drugs on a regular basis. We excluded patients with a main diagnosis of dementia, mental retardation, developmental disorder, or epilepsy. A total of 276 eligible patients were recruited to participate in this study and were informed about this study by research team members or research collaborators who were not involved in the patients’ medical care. Potential participants were clearly informed that their psychiatrists could not see their answers to the STAR-J-P or other instruments, and only patients who consented to participate in this study answered the questionnaire. A subset of the participants completed the STAR-J-P 2 to 4 weeks after its initial administration to permit an evaluation of test-retest reliability. The psychiatrists who regularly provided outpatient treatment to study participants answered the STAR-J-C and provided their own socio-demographic characteristics and the clinical characteristics of each participant (outpatient survey).

To cross-validate the factor structure extracted from an exploratory factor analysis (EFA), we performed a confirmatory factor analysis (CFA). The CFA used a dataset from a research study conducted to investigate the degree of subjective personal agency among individuals with schizophrenia who were participating in assertive community treatment (ACT) at 18 different institutions (Yamaguchi et al, in preparation). Participant eligibility criteria of that study were as follows: 1) diagnosed with schizophrenia and receiving ACT from institutions that participated in the study in December 2017, 2) aged 20 years or older, and 3) capable of providing consent to participate in the study. A total of 252 users and 91 case managers were recruited; they received information about the study, and only those who voluntarily consented to participate answered the questionnaire administered in the present study (ACT survey).

This study was approved by the Research Ethics Committee at the National Center of Neurology and Psychiatry (nos. A2016-044, A2017-063).

### Measurements

#### STAR

STAR, which was developed by McGuire-Snieckus et al. (12), is a scale used to assess the therapeutic relationship between patients and clinicians in community mental health care settings. This scale has two versions, the patient version (STAR-P) and the clinician version (STAR-C). The patient and clinician rate their therapeutic relationship with each other using STAR-P or STAR-C, respectively. Both STAR-P and STAR-C contain 12 items and each item is rated on a 5-point Likert scale, with answers ranging from 0 to 4. The original versions of STAR-P and STAR-C each contain three subscales: for STAR-P, positive collaboration, positive clinician input, and non-supportive clinician input; and for STAR-C, positive collaboration, emotional difficulties, and positive clinician input. The original version of STAR was reported to have acceptable internal consistency, test-retest reliability, and factorial validity (12).

We translated the original version of STAR into Japanese. Back-translation was conducted by a bilingual speaker of Japanese and English. The back-translated scale was confirmed by the author of the original version of STAR. Finally, the detailed wording of the Japanese version of STAR (STAR-J) was adjusted through consultation with community mental health care and outpatient service users for STAR-J-P and psychiatrists for STAR-J-C. During this process, we performed minor adjustments of the Japanese translation to improve the concordance with the original version, clarity of the questionnaire items, and readability.

#### Other Measurements

To examine the convergent validity of STAR-J, we used three scales to assess client satisfaction with their outpatient service, empowerment, and medication adherence. The Japanese version of Client Satisfaction Questionnaire 8-item (CSQ-8-J), originally developed in the USA, was employed to measure client satisfaction (13, 14). The internal consistency and convergent validity of the CSQ-8-J was confirmed in a previous study (14). We used the Boston University Empowerment Scale (BUES) to assess empowerment (15). The Japanese version was developed by Hata et al. (16), and its internal consistency, test-retest reliability, and convergent validity were confirmed. Finally, the Medication Adherence Scale was used to assess medication adherence (17); the internal consistency, convergent validity, and factorial validity of this scale have been confirmed. We hypothesized that STAR- J-P and STAR-J-C scores would be positively correlated with those of CSQ-8, BUES, and the Medication Adherence Scale.

### Characteristics

We asked participants in an outpatient survey about their gender, age, educational status, marital status, living status, and hospitalizations and employment during the previous 6 months. The participating psychiatrists provided information about each participant’s main diagnosis and the presence of coexisting disorders (developmental disorder and mental retardation), as well as the duration of outpatient services provided by the primary psychiatrist. In addition, we collected sociodemographic information for all participating psychiatrists, including age, gender, years of experience as both a medical doctor and a psychiatrist, and educational status. In the ACT survey, we asked participants about their gender, age, educational status, marital status, living status, and hospitalizations and employment during the previous 6 months. The ACT staff members provided information about how long each participant had received ACT services provided by the individual case managers. In addition, we collected sociodemographic information for all participating ACT staff members, including their gender, age, specialty, and years of experience as both psychiatric specialists and as ACT staff members.

### Statistical Analysis

An EFA with geomin rotation was conducted using outpatient survey data to investigate the factor structure of STAR-J. The number of factors was determined based on scree plots and parallel analysis. To determine which items belonged to each factor, we extracted items if they loaded ≥0.4 and showed significant loading on the factor. After the EFA, to confirm cross-validity, we performed a CFA using data from the ACT survey. For estimation in both EFA and CFA, the responses for each item were assumed to be ordinal variables, and the robust weighted least squares method was used due to the highly skewed distribution of each STAR-J item. However, parallel analysis was conducted using the maximum likelihood method because the statistical software could not perform parallel analysis using the robust weighted least squares method. Regarding the CFA, the fit of the model with the data was examined by the chi-squared statistic (CMIN), root mean square error of approximation (RMSEA), comparative fit index (CFI), and Tacker-Lewis fit index (TLI). According to conventional criteria, a good fit would be indicated by CMIN/df < 2, RMSEA < 0.05, CFI > .97, and TLI > .97, while CMIN/df < 3, RMSEA < 0.08, CFI > .95, and TLI > .95 demonstrate an acceptable fit (18).

After confirming the factor structure, we examined internal consistency and convergent validity. Additionally, test-retest reliability was examined for STAR-J-P. To evaluate internal consistency and test-retest reliability, Cronbach’s alpha and intra-class correlation coefficients (ICC) were calculated, respectively. In terms of convergent validity, Spearman’s correlation coefficients were calculated to determine if each of STAR-J-P and STAR-J-C had a positive correlation with CSQ-8-J, BUES, and Medication Adherence Scale. These procedures for confirming reliability and validity were conducted using the outpatient survey data.

Statistical analyses for EFA and CFA were performed using Mplus version 8 (19). Other analyses were conducted using Stata 15.

## Results

### Study Participants

#### Outpatient Survey

We obtained consent for participation from 165 patients (response rate: 59.78%), 20 of whom also consented to answer STAR-J-P twice for determination of test-retest reliability. After 12 participants were excluded due to missing STAR-J-P values and 14 were excluded due to coexistence of mental retardation or developmental disorder, a total of 139 participants (50.36%) were included in the analyses of exploratory factor structure, internal consistency, and convergent validity. In addition, a subset of 17 participants who responded to the questionnaire twice was used for calculation of test-retest reliability.

Table 1 shows participants’ characteristics. Seventy-two (51.80%) were male, and the mean age was 46.02 (SD = 14.12) years. Over half the participants were never married, and over 70% of them lived with their families. Over the past 6 months, 21 (15.11%) individuals had been hospitalized and 62 (44.60%) had been employed. Approximately half of the participants were diagnosed with schizophrenia. The median duration of services received from the primary psychiatrist was 29 months (range: 1–376 months).

**Table 1:** Characteristics of participants in the outpatient survey.

		n/Mean	%/SD
Gender	Male	72	51.80
	Female	67	48.20
Age (years)		46.02	14.12
Education	Junior high school	17	12.23
	High school	41	29.50
	Vocational school	25	17.99
	Junior college	11	7.91
	Undergraduate	40	28.78
	Graduate school	2	1.44
	Other	3	2.16
Marital status	Never married	75	53.96
	Married	48	34.53
	Divorced/bereaved	16	11.51
Living status	Living with family	106	76.26
	Living with others	1	0.72
	Living alone	27	19.42
	Living in cohabitation facilities	3	2.16
	Living in other facilities	2	1.44
Hospitalization during the previous 6 months		21	15.11
Employment during the previous 6 months		62	44.60
Diagnosis	Schizophrenia	68	48.92
	Depression	21	15.11
	Bipolar disorder	19	13.67
	Neurotic, stress-related, and somatoform disorders	22	15.83
	Eating disorders	2	1.44
	Personality disorders	6	4.32
	Other	1	0.72
*Duration of services received from the primary doctor (months)*		*Range: 1–376; median: 29*	

A total of 10 psychiatrists participated in this study. Their mean age was 46.20 years (SD = 12.79), and two (20.00%) were female. The mean duration of experience was 19.50 (SD = 13.05) years as a medical doctor and 13.95 (SD = 12.15) years as a psychiatrist.

#### ACT Survey

We obtained consent for participation from 197 patients (response rate: 78.17%). After two participants were excluded for not completing the questionnaire, 195 participants (77.38%) were included in the CFA.

Table 2 shows the characteristics of the ACT survey participants. One hundred and seven (54.87%) were male, and the mean age was 48.59 (SD = 11.85) years. Around 80% of the participants were never married, and over 40% of them lived alone. Over the previous 6 months, 25 (12.82%) individuals had been hospitalized and 19 (9.74%) had been employed. The median duration of services received from the individual case manager was 28 months (range: 2–396 months).

**Table 2:** Characteristics of participants in the ACT survey.

		n/Mean	%/SD
Gender	Male	107	54.87
	Female	88	45.13
Age (years)		48.59	11.85
Education	Junior high school	43	22.05
	High school	97	49.74
	Vocational school	9	4.62
	Junior college	9	4.62
	Undergraduate	35	17.95
	Graduate school	1	0.51
	Other	1	0.51
Marital status	Never married	159	81.54
	Married	9	4.62
	Divorced/bereaved	27	13.85
Living status	Living with family	69	35.38
	Living alone	83	42.56
	Living in group home or other facilities	43	22.05
Hospitalization during the previous 6 months		25	12.82
Employment during the previous 6 months		19	9.74
*Duration of services received from the case manager (months)*		*Range: 2-396; median: 28*	

Ninety-one case managers participated in this study. Their mean age was 41.75 years (SD = 10.42), and 38 (41.76%) were male. Approximately half (n = 43, 47.25%) of the participating staff members were nurses, 23 (25.27%) were occupational therapists, 23 (25.27%) were psychiatric social workers, one (1.10%) was a clinical psychologist, and one (1.10%) was a psychiatrist. Their mean duration of psychiatry-related employment experience was 176.51 (SD = 116.03) months as a psychiatric specialist and 65.79 (SD = 62.27) months as an ACT staff member.

### Factor Structure

**Figure 1:**
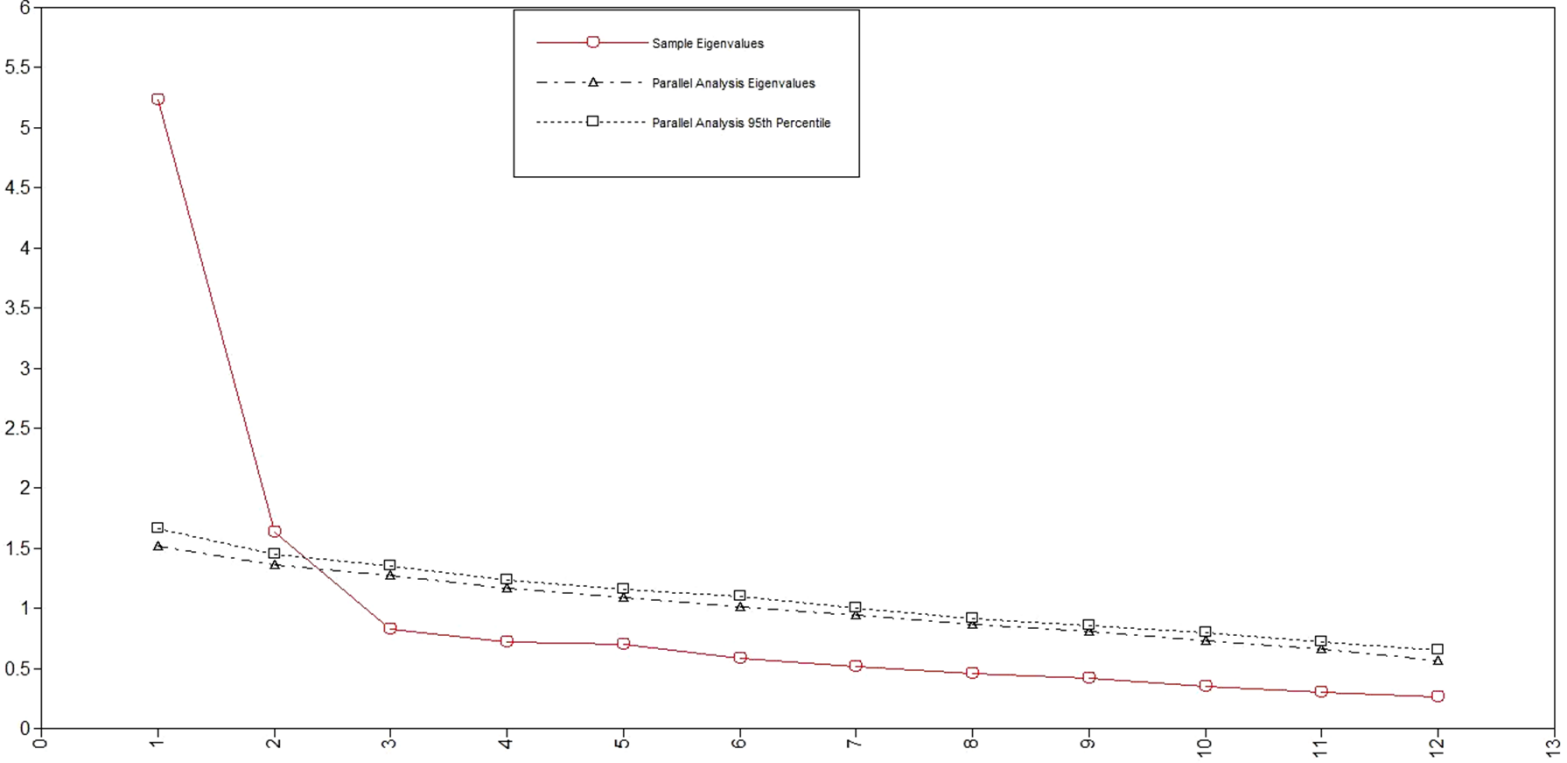
Scree plots and parallel analysis for STAR-J-P.

**Figure 2:**
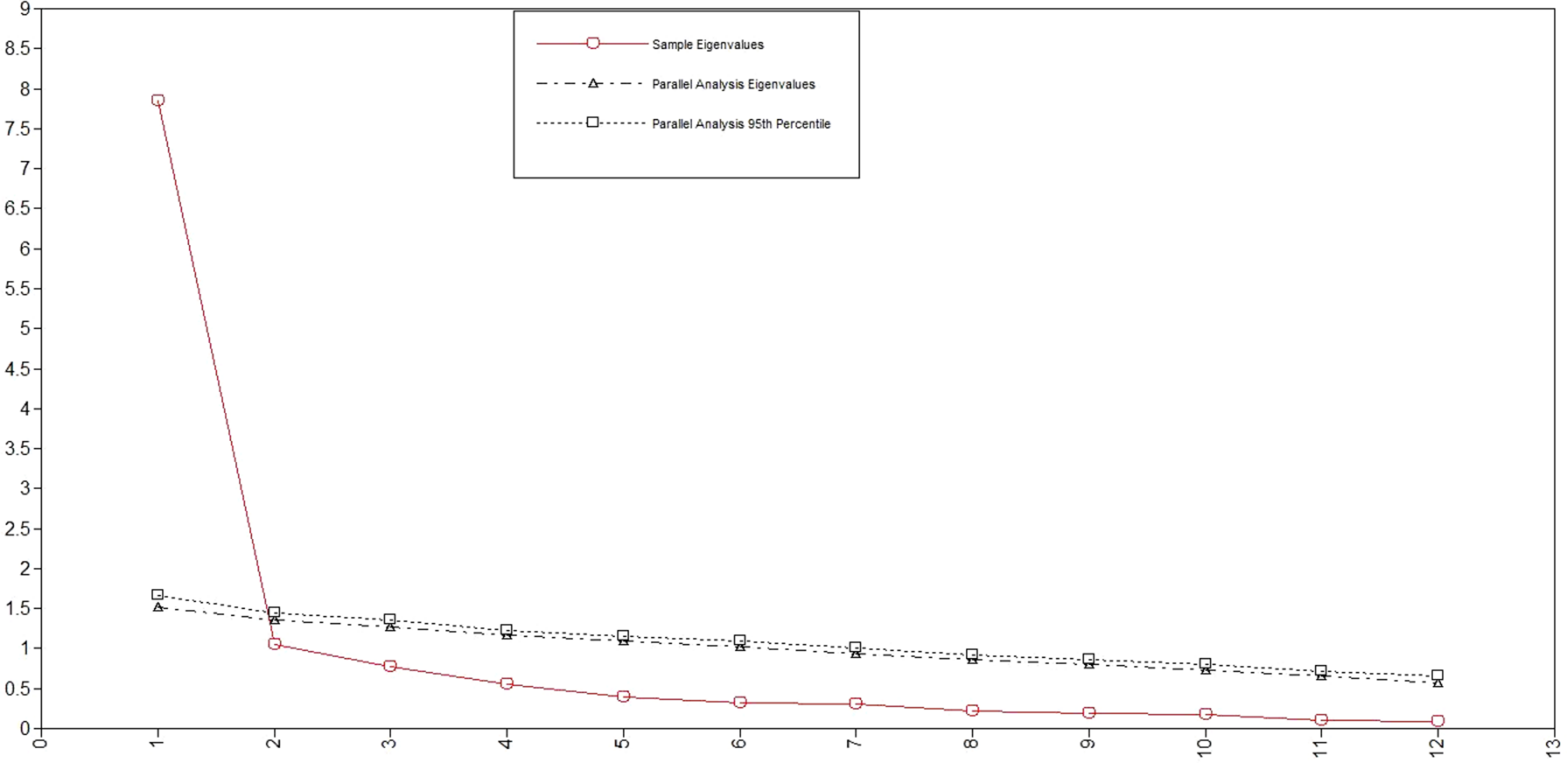
Scree plots and parallel analysis for STAR-J-C.

Factor loadings from the EFA and the means and SD for the STAR-J-P and STAR-J-C items are shown in Tables 3 and 4, respectively. Scree plots for both STAR-J-P and STAR-J-C and parallel analysis suggested a two-factor structure for STAR-J-P and a one-factor structure for STAR-J-C (**Figures 1** and **2**). When a one-factor structure was employed, the factor loading values for all STAR-J-P items were ≥0.4, except for item #4. When a two-factor structure was employed, the factor loading values for all STAR-J-P items were ≥0.4 on either factor. When a three-factor structure was employed, only item #2 showed a factor loading value ≥0.4 for factor 2, but item #2 also showed a factor loading value ≥0.4 for factor 1. Since it was unlikely that STAR-J-P had a three-factor structure, and also considering the results of scree plot and parallel analysis, we employed the one- and two- factor structure for subsequent analysis. Conversely, when a one-factor structure was employed, the factor loading values for all STAR-J-C items were ≥0.4, and we therefore employed all the items. Regarding the two-factor structure of STAR-J-C, only items #6 and #9 showed factor loading values ≥0.4 for factor 2. However, it is pointed out that a factor with 2 variables is only considered reliable when the variables are highly correlated with each another (r > .70) but fairly uncorrelated with other variables (20). The Spearman’s correlation coefficient of items #6 and #9 was 0.391. Since it was unlikely that STAR-J-C had a two-factor structure, and also considering the results of scree plot and parallel analysis, we employed only the one-factor structure for subsequent analysis.

**Table 3:** Factor loadings for STAR-J-P items.

item	1-factor model	2-factor model		3-factor model			Mean	SD
**#1**	**0.759**	**0.761**	0.001	**0.756**	-0.003	0.029	2.957	1.028
**#2**	**0.798**	**0.776**	0.049	**0.609**	**0.510**	0.012	3.324	0.818
**#3**	**0.871**	**0.893**	-0.034	**0.788**	0.299	-0.050	3.295	0.838
#4*	0.334	-0.018	**0.635**	-0.001	-0.090	**0.654**	3.475	0.950
**#5**	**0.820**	**0.816**	0.011	**0.741**	0.221	0.008	3.273	0.788
**#6**	**0.787**	**0.725**	0.136	**0.728**	-0.069	0.182	3.108	0.953
#7*	**0.451**	0.107	**0.659**	0.080	0.045	**0.663**	3.388	1.060
**#8**	**0.768**	**0.764**	0.012	**0.751**	0.010	0.041	3.115	0.941
#9*	**0.467**	0.001	**0.897**	-0.012	0.005	**0.896**	3.604	0.777
**#10**	**0.678**	**0.679**	0.004	**0.658**	0.051	0.017	2.986	1.083
**#11**	**0.846**	**0.874**	-0.051	**0.967**	-0.261	-0.003	3.007	0.936
**#12**	**0.767**	**0.798**	-0.054	**0.779**	0.020	-0.026	2.971	1.049

*reverse item.

Items which loaded ≥0.4 and showed significant loading on the factor are shown in bold.

**Table 4:** Factor loadings for STAR-J-C items.

item	1-factor model	2-factor model		Mean	SD
**#1**	**0.957**	**0.957**	0.002	2.827	0.816
**#2**	**0.980**	**1.099**	-0.192	2.727	0.915
**#3**	**0.920**	**0.897**	0.039	3.079	0.682
#4*	**0.643**	**0.419**	0.331	3.799	0.484
**#5**	**0.986**	**1.016**	-0.055	2.842	0.819
#6*	**0.601**	0.006	**0.874**	3.799	0.527
**#7**	**0.878**	**0.741**	0.217	2.295	0.936
**#8**	**0.910**	**1.009**	-0.156	2.554	1.001
#9*	**0.642**	0.357	**0.450**	2.799	0.886
**#10**	**0.852**	**0.766**	0.140	2.899	0.705
**#11**	**0.932**	**0.872**	0.097	2.568	0.877
**#12**	**0.975**	**0.932**	0.071	2.835	0.795

*reverse item.

Items which loaded ≥0.4 and showed significant loading on the factor are shown in bold.

Because of the insufficient factor loading value of item #4, the CFA of the one-factor model of STAR-J-P was conducted with 11 items. The one-factor model of STAR-J-P showed the following model fit indices: CMIN/df = 7.440 (p < 0.000), RMSEA = 0.182 (90%CI: 0.164-0.200), CFI = 0.893, and TLI = 0.867. However, the path coefficients from the factor to items #7 and #9 were <0.4. After these items were excluded from the model, the model fit indices for the one-factor model with nine items were CMIN/df = 4.346 (p < 0.001), RMSEA = 0.131 (90%CI: 0.107–0.156), CFI = 0.962, and TLI = 0.950, and all path coefficients were >0.4. The CFA of the two-factor model of STAR-J-P for all 12 items showed the following model fit indices: CMIN/df = 2.313 (p < 0.001), RMSEA = 0.082 (90%CI: 0.063-0.101), CFI = 0.975, and TLI = 0.969 (**Table 5**). We tested the original three-factor model of STAR-J-P, but the model estimation was not normally terminated because the coefficients were not positively definite. Among these three models, the model fit indices were best for the two-factor model. Factor 1 was loaded by items such as “My clinician speaks with me about my personal goals and thoughts about treatment” (#1), “My clinician and I are open with one another” (#2), “My clinician and I share a trusting relationship” (#3), “My clinician and I share an honest relationship” (#5), “My clinician and I work towards mutually agreed upon goals” (#6), “My clinician and I have established an understanding of the kind of changes that would be good for me” (#8), “My clinician seems to like me regardless of what I do or say” (#10), “We agree on what is important for me to work on” (#11), and “I believe my clinician has an understanding of what my experiences have meant to me” (#12). We considered these items as reflective of Positive Clinician Input and Collaboration. Factor 2 was loaded by items such as “I believe my clinician withholds the truth from me” (#4), “My clinician is stern with me when I speak about things that are important to me and my situation” (#7), and “My clinician is impatient with me” (#9). We considered these items as reflective of Non-supportive Clinician Input.

**Table 5:** Model fit indices of CFA of STAR-J-P.

	CMIN/df	RMSEA	CFI	TLI
**1-factor with 11 items**	7.440	0.182	0.893	0.867
**1-factor with 9 items**	4.346	0.131	0.962	0.950
**2-factor**	2.313	0.082	0.975	0.969

The CFA of STAR-J-C for the one-factor structure showed the following model fit indices: CMIN/df = 2.808 (p < 0.001), RMSEA = 0.096 (90%CI: 0.078-0.115), CFI = 0.985, and TLI = 0.982. We also tested the original three-factor model for STAR-J-C, and the model fit indices were: CMIN/df = 2.214 (p < 0.001), RMSEA = 0.079 (90%CI: 0.059-0.099), CFI = 0.990, and TLI = 0.988 (**Table 6**).

**Table 6:** Model fit indices of CFA for STAR-J-C.

	CMIN/df	RMSEA	CFI	TLI
**1-factor model**	2.808	0.096	0.985	0.982
**original 3-factor model**	2.214	0.079	0.990	0.988

### Reliability

We tested the reliability of STAR-J-P with a two-factor structure and of STAR-J-C with a one-factor structure. Cronbach’s alphas of STAR-J-P factor 1, factor 2, and STAR-J-C were 0.897, 0.645, and 0.949, respectively. In terms of the test-retest reliability of STAR-J-P, the ICC was 0.765 (95% CI: 0.462–0.908, p < 0.001) for factor 1 and 0.630 (95% CI: 0.219–0.849, p = 0.003) for factor 2.

### Convergent Validity

Spearman’s correlation coefficients between the STAR-J-P two-factor model, STAR-J-C, CSQ-8, BUES, and the Medication Adherence Scale are shown in **Table 7**. STAR-J-P factor 1 showed significant and positive correlations with CSQ-8 (ρ = 0.648, p < 0.001), BUES (ρ = 0.283, p = 0.001), and the Medication Adherence Scale (ρ = 0.508, p < 0.001). STAR-J-P factor 2 showed significant and positive correlations with CSQ-8 (ρ = 0.238, p = 0.005) and the Medication Adherence Scale (ρ = 0.347, p < 0.001) but not with BUES (ρ = 0.088, p = 0.317). STAR-J-C demonstrated a significant and positive correlation only with BUES (ρ = 0.207, p = 0.017). Either STAR-J-P factor 1 or factor 2 and STAR-J-C were not significantly correlated with each other (ρ = 0.158, p = 0.063 for factor 1; ρ = 0.060, p = 0.484 for factor 2).

**Table 7:** Spearman’s correlation coefficients between STAR-J-P, STAR-J-C, CSQ-8, BUES, and Medication Adherence Scale.

		1		2		3		4		5		6
1	STAR-J-P factor1	1.000										
2	STAR-J-P factor2	0.355	***	1.000								
3	STAR-J-C	0.158		0.060		1.000						
4	CSQ-8	0.648	***	0.238	**	0.039		1.000				
5	BUES	0.283	**	0.088		0.207	*	0.121		1.000		
6	Medication Adherence Scale	0.508	***	0.347	***	0.098		0.527	***	0.356	***	1.000

*p < 0.05,**p< 0.01,***p < 0.001.

## Discussion

In this study we evaluated the psychometric properties of the Japanese version of STAR, and specifically investigated its reliability and validity in Japanese psychiatric service users and specialists. In factor analyses, STAR-J-P demonstrated a two-factor structure and STAR-J-C demonstrated a one-factor structure. Both STAR-J-P and STAR-J-C showed good psychometric properties, with relatively high Cronbach’s alphas (> 0.85), except for STAR-P factor 2, and good ICCs (0.765 and 0.630 for factors 1 and 2 of STAR-J-P, respectively), as well as convergent validity with other measures.

### Factor Structure

The present study identified a two-factor structure for STAR-J-P and a one-factor structure for STAR-J-C. The factor loading values of each item in both scales were ≥0.4, and the model fit indices were good or acceptable. Although the RMSEA values for STAR-J-P and STAR-J-C were >0.08, this criterion was previously shown to have little empirical support (21), and values <0.10 have been interpreted as acceptable in several studies (22–25). Our findings suggest that STAR-J-P and STAR-J-C have good factor structures as a two-factor model and one-factor model, respectively, even though the original versions of both STAR-P and STAR-C were reported as three-factor models.

The factor structure for the original version of STAR was extracted by principal component analysis; this data reduction method differs from factor analysis, which aims to reveal latent variables from observational variables (26). One reason for the different factor structures of the original and Japanese versions of STAR may be the distinct analytical methods used in each study. Cultural differences between Japan and the UK may also have contributed to different factor structures between this study and the past study. Factor 2 consisted of only reverse items #4, #7, and #9, which pertained to non-supportive clinician input, while factor 1 was related to positive clinician input and collaboration and contained the items that were divided into two factors in the original study. EFA identifies latent factors based on the similarity of items. The effects of “positive clinician input and collaboration” and “non-supportive clinician input” on the therapeutic relationship might be more heterogeneous in Japanese clinical settings than in the United Kingdom. Therefore, items about positive clinician input and collaboration might be loaded on the same factor. Indeed, non-supportive clinician input might be interpreted differently in Japan compared to western countries. Telling the truth to patients is considered essential from a bioethical point of view, but in Japan, the truth may be withheld in certain situations. For example, clinicians often tend to conceal negative prognoses from patients with advanced or end-stage cancer (27, 28). Furthermore, in Japanese settings, being honest means sharing one’s sincere feelings with another person, but it is not always necessary to disclose the truth about patients’ medical conditions (29). These viewpoints about truth-telling and honesty in medical services may be widely shared among Japanese laypeople, including the participants in outpatient survey used for EFA. Hence, when considering the therapeutic relationship in a Japanese context, Japanese clinicians’ attitudes toward telling the truth to the patients might be limited. For the participants in this study, the therapeutic relationship comprises both the positive clinician input and collaboration as well as non-supportive clinician input; however, it differently influences service users’ empowerment, service satisfaction, and medication adherence. These cultural differences between Japan and the United Kingdom led to variations in conceptualizing the patient-clinician therapeutic relationship. Additionally, the participating clinicians in outpatient survey data that used for EFA consisted only of psychiatrists, while the United Kingdom study included social workers, nurses, psychologists, and occupational therapists. Japanese participants may have more difficulty negatively rating their therapeutic relationships with psychiatrists than with other psychiatric professionals, since culturally they are likely to rely on their psychiatrists or medical doctors (30). These cultural and disciplinary differences might have affected the factor structure of STAR-J-P extracted by EFA.

Regarding the results of CFA of STAR-J-C, the original three-factor model showed better model fit indices than the one-factor model extracted by EFA, although the original model was not extracted by EFA. This discrepancy might be due to the fact that the two datasets had different sample populations. The original version of STAR was developed in a community mental health setting. In this study, for the EFA we recruited participants in outpatient settings and only psychiatrists as staff. On the other hand, the CFA was based on survey data on ACT staff members with a variety of professional backgrounds, and as such, more closely resembles the original study. These sample characteristics might explain why the original factor model was confirmed and showed better model fit indices than the one-factor model extracted by EFA.

### Reliability and Validity

The minimum threshold of Cronbach’s alpha for internal consistency is 0.7 (31). The Cronbach’s alpha values of STAR-J-P factor 1 (α = 0.897) and STAR-J-C (α = 0.949) were satisfactorily high. On the other hand, for STAR-J-P factor 2, the alpha value was relatively low (α = 0.645). However, this could be due to the small number of questions (32). ICC values as measures of test-retest reliability for STAR-J-P (0.765 for factor 1 and 0.630 for factor 2) can be assumed to be acceptable, considering the general criterion that ICC values between 0.5 to 0.75 indicate moderate reliability and 0.75 to 0.90 indicate good reliability (33).

STAR-J-P appeared to have good convergent validity, consistent with conceptual associations between variables reported in past studies (7–10). There was the exception, namely factor 2 and BUES, which might be due to the aforementioned Japanese viewpoint on non-supportive clinician input. On the other hand, STAR-J-C showed significant correlation only with BUES. However, all the scales used to evaluate convergent validity were self-reported by patients rather than completed by psychiatrists. As pointed out in a relevant review, the therapeutic relationship may be perceived differently by patients on the one hand and psychiatrists or clinicians on the other (4). This may explain why STAR-J-C was unexpectedly correlated only with BUES and also why there was no significant correlation between STAR-J-P and STAR-J-C.

### Limitations

The present study has several limitations. First, as discussed above, the clinicians in the outpatient survey included only psychiatrists and no other types of professionals. To better generalize the findings of this study, future research should test the psychometric properties of STAR-J in broader settings such as other mental health facilities. However, we tested cross-validity by conducting a CFA using a sample from a different population. Therefore, the generalizability of the factor structure was supported to a certain extent. Second, the sample size of this study was moderate to achieve stable results when conducting EFA and CFA. Therefore, these results should be assumed as preliminary. Third, each participating psychiatrist and case manager evaluated their relationships with multiple patients, which resulted in correlated data that were not controlled in this study. Finally, test-retest reliability was investigated only for STAR-J-P, and this property remains unclear for STAR-J-C. Future studies should address these problems to enhance the evidence on STAR-J.

## Conclusions

We developed STAR-J and examined its psychometric properties in Japanese psychiatric service users and clinicians. Through factor analyses, we found that STAR-J-P demonstrated a two-factor structure and STAR-J-C demonstrated a one-factor structure. Internal consistency and convergent validity for both STAR-J-P and STAR-J-C and test-retest reliability for STAR-J-P were acceptable except for no significant correlation of STAR-J-P factor 2 with empowerment and of STAR-J-C with users’ service satisfaction and medication adherence. Overall, STAR-J should be considered as a useful instrument for evaluating the therapeutic relationship in Japanese community mental health settings. Further studies must investigate validity and applicability to various mental health care service settings and professionals.

## Ethics Statement

All participants were informed about this study and only patients who consented to participate in this study answered the questionnaire. The authors assert that all procedures contributing to this work comply with the ethical standards of the relevant national and institutional committees on human experimentation and with the Helsinki Declaration of 1975, as revised in 2008. This study was approved by the Research Ethics Committee at the National Center of Neurology and Psychiatry (nos. A2016-044, A2017-063).

## Author Contributions

AM, SY, and US designed the study protocol, and AM, SY, US, and TS coordinated the data collection. AM and SY performed statistical analysis and drafted the manuscript. US, TS and CF helped to draft the manuscript. CF participated in coordination and supervised the study. All authors read and approved the final manuscript.

## Funding

This work was supported by the Japan Agency for Medical Research and Development (AMED) [grant number 16dk0307059h0001] (a co-production project to develop a practical guidance for patient-centred and life-oriented recovery of schizophrenia).

## Conflict of Interest Statement

The authors declare that the research was conducted in the absence of any commercial or financial relationships that could be construed as a potential conflict of interest.
